# If you build it, they will stay! Sustaining evidence-based practices for the treatment of substance use disorders through investment in workforce retention

**DOI:** 10.3389/fpubh.2026.1939991

**Published:** 2026-09-04

**Authors:** Michael J. Chaple, Sara Becker, Thomas E. Freese, Bryan Hartzler, Rosemarie Martin, Todd Molfenter, Beth A. Rutkowski

**Affiliations:** 1 Division on Substance Use Disorders, New York State Psychiatric Institute, New York, NY, United States; 2 Department of Psychiatry, Columbia University Medical Center, New York, NY, United States; 3 Center for Dissemination and Implementation Science, Northwestern University Feinberg School of Medicine, Chicago, IL, United States; 4 Integrated Substance Use and Addiction Programs, University of California, Los Angeles, Los Angeles, CA, United States; 5 University of Washington School of Medicine, Seattle, WA, United States; 6 Population and Quantitative Health Sciences, University of Massachusetts Chan Medical School, Worcester, MA, United States; 7 Department of Industrial and Systems Engineering, University of Wisconsin-Madison, Madison, WI, United States

**Keywords:** organizational change, staff retention, substance use disorders, technical assistance, workforce development

## Abstract

Much attention has been paid to the persistent gap between the development of effective, evidence-based practices (EBPs) for substance use disorders (SUDs) and their routine delivery in real-world clinical and community settings. The struggle to routinely implement and sustain EBPs with fidelity has been attributed to a combination of systemic, organizational and provider-level barriers. These barriers are compounded by high rates of staff turnover, as behavioral health systems must continuously reinvest in professional development despite their limited resources. Intermediary Purveyor Organizations (IPOs) such as the Addiction Technology Transfer Centers (ATTCs) have been engaged to support capacity building within agencies or systems to implement and sustain EBPs but must also contend with these challenges. As we expand implementation science efforts to explore the contextual factors that influence EBP implementation, including the impact of IPOs, it is important to consider that failure to improve staff turnover rates will only undermine these scientific investments. From the perspective of purveyors of technical assistance, the authors first reflect on the landscape of staff turnover, then offer system- and organization-level strategies for improving staff retention rates.

## Introduction

Research has consistently shown that clinically-based evidence-based practices (EBPs) for the treatment of substance use disorders (SUDs) are underutilized in clinical settings (1). Low rates of utilization are well-documented for front-line treatments such as medication for opioid use disorder (MOUD) (2), which is only prescribed to about 20–25% of people who need it, and contingency management (CM) (3, 4), which remains the least implemented of the empirically based treatments (5). Self-reported use of evidence-based behavioral techniques such as Cognitive Behavioral Therapy (CBT) and Motivational Interviewing (MI) tend to be very high but rarely is there any evidence that practice is supported by a manual, guidelines or other forms of technical assistance (TA) that would help to assure fidelity (6, 7). Thus, while effective treatments exist for SUDs, there is clear consensus that not only are they not widely practiced but when practiced, accurately determining the fidelity of innovation delivery is extremely difficult (8, 9).

It has been suggested that EBP scale-up has been limited largely because of weaknesses in the distribution system for bringing treatment innovations to the attention of practitioners and into practice settings (10). Intermediary Purveyor Organizations (IPOs) are designed to address these weaknesses. IPOs are organizations that are external to the clinical sites that need to implement EBPs and provide TA to support implementation. As a result, IPOs build capacity within agencies and systems to implement and sustain best practices (11–13), work to identify the best intervention fit for a specific organization’s needs, link resources to end user clinicians and administrators, and provide coaching about implementation processes for specific EBPs (14). IPOs fill a critical gap of facilitating EBP adoption when current environmental conditions are yielding low adoption rates. The Addiction Technology Transfer Center (ATTC) Network is a prime example as a set of linked, regionally defined IPOs, funded by the Substance Abuse and Mental Health Services Administration (SAMHSA) to support the adoption and implementation of EBPs for SUDs among the addictions workforce (15). ATTC grantees have purview over all fifty states, the District of Columbia, U. S. Territories and Freely Associated States, leveraging a wide array of TA strategies to provide implementation support. While research has shown that training can improve clinicians’ understanding of and confidence in treating SUD (16), stand-alone educational workshops rarely produce sustainable practice change without ongoing TA support, clinical consultation, and fidelity monitoring (17, 18). This work has consistently demonstrated that ongoing TA is vital for EBP implementation (19, 20), yet the routine provision of TA is complicated by organizational, systemic, and financial barriers that affect engagement and effectiveness.

Barriers to EBP implementation and sustainment span multiple levels. According to the Consolidated Framework for Implementation Research (CFIR), implementation success is dependent upon characteristics of the: intervention (e.g., specialized staff competence required, treatment duration, and cost); practitioners (e.g., time available, knowledge and skills, beliefs about the nature and effectiveness of treatment options, alignment with values, perceived self-efficacy); clients (e.g., health literacy, needs, resources, attitudes, readiness for change); inner setting (e.g., program size, length of time in operation, physical space, organizational culture, leadership engagement, support for professional development, resource limitations), and outer setting (e.g., societal stigma toward clients and treatments, limited reimbursement, regulatory hurdles) (21). It is also well-established that barriers to EBP implementation are more complex in the SUD treatment field than in many areas of the physical health field due to pervasive stigma, structural disparities in reimbursement, and regulatory barriers that impact care delivery (22, 23).

In this viewpoint, drawing on our vantage point as leaders within the ATTC Network who have collectively been providing TA for over three decades, we argue that one of the greatest threats to the sustainability of SUD innovations are the provider and inner setting factors as they relate to staff turnover. As cutting-edge TA purveyors, we first reflect on the landscape of staff turnover and then offer system- and organization-level strategies for improving staff retention rates.

### The challenge of staff turnover

In the SUD treatment field, the annual turnover rate can reach as high as 50 percent (24), with the implications of turnover likely to be especially profound when implementing EBPs (25). This high rate of turnover destabilizes SUD treatment organizations, creating a crisis that undermines quality care, drains resources, and threatens the very foundation of clinical services. Studies have shown that staff in high turnover settings report higher demands, and lower support and morale within their organization, even after controlling for factors such as decreasing budgets or increased census (26). Because EBPs are highly structured and require specialized training, staff turnover forces behavioral health systems that are integrating EBPs to reinvest in professional development. For instance, a prior review of 13 randomized clinical trials evaluating EBPs found that all 13 had to conduct two or more staff retraining sessions to accommodate turnover (27). If supervisors and other experienced clinicians leave, this can negatively impact the quality of implementation oversight and the potential for ongoing professional development, while also impeding an organization’s ability to onboard and train new hires. A recent scoping review found that the most frequent impacts of staff turnover on EBP implementation efforts included loss of organizational knowledge, increased need for training, increased costs and financial stress, and reduced EBP fidelity (28). Taken together, these consequences erode the implementation climate and undermine a stable learning environment that is necessary to support EBPs.

When it comes to SUD treatment, we know that most staff leave voluntarily, typically due to job dissatisfaction (22, 29). Potential specific reasons for staff turnover include, though are not limited to: lack of adequate compensation and benefits; burnout, stress, and compassion fatigue due to relentless productivity demands and exposure to client trauma; ineffective leadership, poor organizational communication, or a lack of consistent clinical supervision; having to navigate excessive administrative burdens such as paperwork, regulatory requirements, and insurance authorizations; limited opportunity for advancement (i.e., salary increases, promotions, and professional certifications); and lack of engagement in the work due to limited opportunities for input or inability to identify impact on client outcomes (30–32). Because the majority of staff turnover is voluntary, with reasons for leaving often being job-related, staff turnover is far from inevitable. Yet limited research has examined specific factors promoting staff retention and, with a few exceptions (33), reviews have rarely provided concrete recommendations to address turnover.

### Innovative strategies to improve staff retention

Given the dearth of research, there is limited understanding of effective workforce retention strategies for the SUD treatment field (34). However, there is an important opportunity to draw upon IPO experience to identify strategies that would likely result in improved job satisfaction for SUD professionals in diverse service settings. Like the way IPOs support EBP implementation, they can also support implementation of workforce retention strategies. Given their historical emphasis on forging longstanding partnerships with single-state authorities, regional stakeholders, and community health organizations, the ATTCs may be uniquely positioned to observe, track, and promote existing forms of practice-based evidence in this area. In this section, we outline practice-informed strategies that ATTCs and other IPOs can use to promote staff retention, organized into two broad categories: administrative and service strategies.

#### Administrative strategies that may require consultation support

Administrative solutions to workforce retention challenges include strategies to improve the infrastructure that governs the workplace. Strategies in this category typically target employee compensation, clinical workflows, career advancement, and work-life balance. Historically, the ATTC Network has supported the field by raising awareness of these strategies, enhancing knowledge of their impact, providing consultation on policy and practice design, and connecting programs to early adopters to promote peer-to-peer learning. A prior content analysis of ATTC TA events (35) found that approximately 10% were categorized as “organizational management and communication,” the topic that would encompass TA focused on staff retention. These administrative implementation strategies focused on structural factors such as resources, organizational workflows, organizational infrastructure, and incentives.

##### Offer competitive compensation and benefits

Compensation and benefit structures should meet or exceed industry standards, even within budget-constrained environments, as low wages have been identified as one of the strongest factors in SUD workforce turnover (36, 37). While full wage parity with larger health systems will be difficult, incremental improvements paired with flexible benefits can demonstrate strong organizational commitment to workforce equity. Structured wage supplement models provide bonuses or raises for employees who meet specific recovery milestones within their caseload or reach tenure benchmarks within the program (38). Benefits can make up approximately one third of employees’ overall compensation and are often as valuable as salaries. Benefits packages can be enhanced by expanding health insurance, retirement plans, contributions to student loan repayment, and support for student loan forgiveness (39). Retirement plans, especially with employer contributions, ensure future security for employees. These strategies have been promoted by the ATTC network (40), including learning collaboratives involving administrative change teams focused on compensation and reward packages.

##### Modernize tools and processes

The administrative burden in operating an SUD treatment facility adversely impacts providers’ ability to deliver SUD services efficiently and effectively (41). Administrative demands in SUD treatment organizations stem from many sources such as accreditation and licensing processes; documentation of care provision; insurance pre-authorizations, follow-up reviews, and ongoing utilization review; managing the complex requirements of various external funding sources; and complying with federal employment requirements and patient information privacy regulations such as the Health Insurance Portability and Accountability Act (HIPAA). These administrative burdens result in providers spending more time on paperwork than on direct patient care (42, 43). To mitigate these burdens, programs can consider ways to streamline workflows. De-implementation checklists can identify common implementation burdens and eliminate requirements that add little value to patients and their care teams (44). Examples include streamlining fax-based work to electronic automated systems and reducing duplicative work (45), simplifying EHR-based workflows (46–48), reviewing the volume of and requirements for prior authorizations (49), automating data collection for any needs that are secondary to clinical care (50), and reassigning routine administrative and clinical work to clinical support staff (50). For example, Hawai’i Pacific Health’s “Getting Rid of Stupid Stuff” project gathered feedback from employes on their experiences with the EHR and solicited nomination of tasks to eliminate; this process resulted in 1,700 nursing hours saved per month across their entire health care system (51). The ATTC network has facilitated learning collaboratives designed to reduce paperwork burden including NIATx leadership academies focused on task elimination.

##### Implement policies that promote work-life balance

Focused policies to prevent burnout and mitigate secondary trauma, as a people-first workplace with clear boundaries produces well-adjusted and more engaged personnel. Within the clinical space, mandatory clinical supervision can provide a safe forum for processing secondary trauma and compassion fatigue and for regularly reviewing provider self-care plans (52, 53). Boundaries can be reinforced through strict protocols for limiting personal contact with participants, supported by centralized systems for handling after-hours calls. In addition, administrators can offer flex time, remote work schedules, telehealth counseling, shift trades, or floating teams to accommodate the personal lives of their clinical workforce (54). Robust paid time off (PTO) policies can be structured to offer generous accrual rates and allow employees to attend medical appointments, therapy, or periods of rehabilitation without financial distress. Employee Assistance Programs (EAPs) that provide specialized behavioral health counseling tailored to the workforce should be actively promoted. Employers should also highlight short-term disability options required by law such as the Family and Medical Leave Act (FMLA) and the Americans with Disabilities Act (ADA). Recovery from SUD is a protected disability that helps to ensure that staff who have their own substance use challenges do not lose their license or employment when they seek help. Collectively, these policies can reduce burnout and turnover intentions, strengthening capacity to sustain EBPs.

##### Support career paths for advancement

Staff development initiatives that support career advancement will help to prevent staff from seeking opportunities elsewhere (55, 56). Mentorship programs help to enhance clinical competencies, support EBPs, mitigate staff burnout, and ultimately equip team members with the skills and resiliency necessary to excel. Career pathing frameworks map more structured routes for career advancement, supported by the provision of targeted professional development, clear credentialing requirements, and a commensurate salary structure. Policies that address credentialing and license reimbursement, tuition assistance and loan repayment will attract and retain qualified and ambitious clinical staff.

#### Service strategies that may require intensive TA support

Strategies implemented directly with program staff to improve skills and foster resilience in the workplace promote staff retention. Strategies in this category focus on organizational culture, leadership development, clinical supervision, employee well-being, and much more. About 18% of all ATTC TA provision has supported the field in this area (35). Examples of intensive TA in this domain include Leadership Academies, communities of practice for clinical supervision, training and coaching to enhance self-care and reduce compassion fatigue, and TA strategies focusing on organizational culture via promotion of person-centered care, stigma reduction, recovery-oriented systems of care, team-based care, holistic treatment approaches, and collaborative change models. These service-level approaches function as implementation strategies aimed at improving implementation climate, learning climate, and provider attitudes and skills.

##### Invest in leadership development

The administrative recommendations put forth previously require strong leadership that is in tune with the multi-level factors affecting staff retention. The ATTC Network has a rich history of leadership development facilitated via intensive learning academies designed to help emerging leaders integrate effective leadership practices into their organizational workflow (57). Although the structure of these initiatives vary by regional ATTC, they generally will: assess for strengths and potential growth areas, offer immersion training that provides in-depth guidance on the latest leadership practices and behavioral health strategies, include expert panels on emerging topics, provide expert coaching and mentorship to support continuous professional development, and facilitate membership in an alumni network that offers opportunities for networking, collaboration, and professional growth. Leaders trained via these initiatives have reported gains in the quality of care along with increased confidence in their leadership potential, organizational growth, priority management, and team empowerment.

##### Enhance clinical supervision competencies

Clinical supervision, distinct from administrative supervision, involves supportive functions intended to create a positive work environment for clinical staff. Intended benefits of clinical supervision include: supporting the development of counseling proficiencies by expanding evidence-based clinical skills; promoting professional advancement by guiding staff toward licensure, credentialing, and other pathways for career progression; and minimizing staff frustration by setting clear expectations, appropriately structuring roles, monitoring and adjusting caseloads, and aligning practices with organizational policies (58). Clinical supervision is also intended as a mechanism for the provision of emotional support whereby counselors can access a safe place to process job-related stressors, including difficult clinical interactions. A recent scoping review found that effective clinical supervision was associated with lower burnout and greater staff retention, and that healthcare professionals perceived inadequate clinical supervision as leading to stress and burnout (59). The ATTC Network has worked extensively to enhance clinical supervision competencies, starting with the Clinical Supervisions Foundations course (60), which provides 30 h of instruction (14 h virtual, 16 h face-to-face) on the essential elements of clinical supervision practice. Many regional ATTCs have supplemented this coursework with ongoing TA support in the form of learning collaboratives, coaching, audit and feedback, and other TA strategies.

##### Proactively address staff wellness

Organizations should also focus on providing supports that indirectly impact staff wellness through the implementation of best practices that positively affect organizational culture. This includes a focus on person-centered care, which helps to shift organizational culture from a focus on compliance to prioritizing safety and communication. Similarly, motivational interviewing (MI) promotes communication techniques that build partnership, trust, and positive interpersonal dynamics, which serve as protective factors against burnout. Support for vicarious trauma can help to address the constant exposure to clients’ trauma that tends to break down resilience and lead to emotional exhaustion. This may be especially impactful for staff that have personally dealt with similar traumas and are at risk of re-traumatization. The ATTC Network has expertise in this area, routinely providing training and TA on person-centered care, MI, trauma-informed care, and the development of coping skills for staff who have lost clients to suicide or overdose. The ATTC Network has also helped to raise awareness of and offer strategies for addressing compassion fatigue and promoting self-care.

## Discussion

Since the ATTCs were established more than three decades ago, we have confirmed in practice what the research has documented: EBPs for SUDs are not widely implemented, and when they are implemented, they are rarely implemented with fidelity. Implementation efforts have been impeded by extensive and complex barriers at the individual, organization and systemic levels, making the support of IPOs such as the ATTCs essential to large-scale implementation of EBPs. In this viewpoint, we aim to elevate workforce retention as a central implementation determinant and call for implementation research on TA models that overtly incorporate retention strategies.

ATTCs appreciate the need for a multi-faceted approach to EBP implementation as stressed by the CFIR model. Specifically, the ATTCs have found in practice that issues that arise due to workforce retention challenges create an unstable environment for EBP implementation. Addressing workforce retention is a multifaceted issue and through the administrative and service strategies described in this paper, a multifaceted approach to creating greater workforce retention is advanced. In sum, developing career ladders, finding innovative compensation strategies, eliminating frustrating work processes, fostering work-life balance, enhancing leadership skills and staff supervision, all of which the ATTC Network has routinely addressed through TA, have proven to be practice-based changes that help to facilitate staff retention.

Although the challenges created by staff turnover have been recognized as a significant barrier to EBP implementation, there has been insufficient emphasis placed on staff retention as a means of creating more fertile ground for successful implementation. As the field invests more heavily in EBP dissemination and implementation, it will be critical to treat workforce retention not as a peripheral HR concern but a central priority. IPOs can support these efforts through targeted and intensive TA focused on administrative and service-level strategies designed to promote retention and mitigate turnover. Partnering with IPOs in this way can help to optimize the growing investment in efforts to bridge the research to practice gap and make it more likely that EBPs for SUD treatment are adopted, delivered with fidelity, and sustained over time.

## Data Availability

The original contributions presented in the study are included in the article/supplementary material, further inquiries can be directed to the corresponding author/s.

## References

[1] McGovern MP Saunders EC Kim E. Substance abuse treatment implementation research. J Subst Abus Treat. (2013) 44:1–3. doi: 10.1016/j.jsat.2012.09.006, 23083972 PMC3718484

[2] Knudsen HK Abraham AJ Roman PM. Adoption and implementation of medications in addiction treatment programs. J Addict Med. (2011) 5:21–7. doi: 10.1097/ADM.0b013e3181d41ddb, 21359109 PMC3045214

[3] Becker SJ DiClemente-Bosco K Rash CJ Garner BR. Effective, but underused: lessons learned implementing contingency management in real-world practice settings in the United States. Prev Med. (2023) 176:107594. doi: 10.1016/j.ypmed.2023.107594 PMC10753028 37385413

[4] Oluwoye O Kriegel L Alcover KC McPherson S McDonell MG Roll JM. The dissemination and implementation of contingency management for substance use disorders: a systematic review. Psychol Addict Behav. (2020) 34:99–110. doi: 10.1037/adb0000487, 31259569 PMC6938576

[5] Petry NM Alessi SM Olmstead TA Rash CJ Zajac K. Contingency management treatment for substance use disorders: how far has it come, and where does it need to go? Psychol Addict Behav. (2017) 31:897–906. doi: 10.1037/adb0000287, 28639812 PMC5714694

[6] Haug NA Shopshire M Tajima B Gruber V Guydish J. Adoption of evidence-based practices among substance abuse treatment providers. J Drug Educ. (2008) 38:181–92. doi: 10.2190/DE.38.2.f, 18724657 PMC2742313

[7] Louie E Barrett EL Baillie A Haber P Morley KC. Implementation of evidence-based practice for alcohol and substance use disorders: protocol for systematic review. Syst Rev. (2020) 9:25. doi: 10.1186/s13643-020-1285-0, 32033587 PMC7007686

[8] Carroll KM. Dissemination of evidence-based practices: how far we've come, and how much further we’ve got to go. Addiction. (2012) 107:1031–3. doi: 10.1111/j.1360-0443.2011.03755.x, 22324509

[9] Finney JW Hagedorn HJ. Introduction to a special section on implementing evidence-based interventions for substance use disorders. Psychol Addict Behav. (2011) 25:191–3. doi: 10.1037/a0023949, 21668084

[10] Kreuter MW Casey CM Bernhardt JM. Enhancing Dissemination through Marketing and Distribution Systems: a vision for public Health. Eds. Brownson R. C. Colditz G. A. Proctor E. K.. Oxford: Oxford University Press (2012).

[11] Franks R Bory C. Who supports the successful implementation and sustainability of evidence-based practices? Defining and understanding the roles of intermediary and purveyor organizations. New Dir Child Adolesc Dev. (2015) 2015:41–56. doi: 10.1002/cad.20112, 26375190

[12] Corcoran T Rowling L Wise M. The potential contribution of intermediary organizations for implementation of school mental health. Adv School Ment Health Promot. (2015) 8:57–70. doi: 10.1080/1754730X.2015.1019688

[13] Franks RP. Role of the intermediary organization in promoting and disseminating best practices for children and youth: the Connecticut center for effective practice. Emotional Behav Disord Youth. (2010) 10:87–93.

[14] Proctor E Hooley C Morse A McCrary S Kim H Kohl PL. Intermediary/purveyor organizations for evidence-based interventions in the US child mental health: characteristics and implementation strategies. Implement Sci. (2019) 14:1–14. doi: 10.1186/s13012-018-0845-3, 30642342 PMC6332855

[15] Addiction Technology Transfer Center Network. Available online at: https://attcnetwork.org/ (Accessed May 23, 2026).

[16] Iheanacho T Bommersbach T Fuehrlein B Arnaout B Dike C. Brief training on medication-assisted treatment improves community mental health clinicians’ confidence and readiness to address substance use disorders. Community Ment Health J. (2020) 56:1429–35. doi: 10.1007/s10597-020-00586-8, 32062717 PMC7429311

[17] Beidas RS Kendall PC. Training therapists in evidence-based practice: a critical review of studies from a systems-contextual perspective. Clin Psychol Sci Pract. (2010) 17:1–30. doi: 10.1111/j.1468-2850.2009.01187.x, 20877441 PMC2945375

[18] Frank HE Becker-Haimes EM Kendall PC. Therapist training in evidence-based interventions for mental health: a systematic review of training approaches and outcomes. Clin Psychol Sci Pract. (2020) 27:e12330. doi: 10.1111/cpsp.12330 PMC8174802 34092941

[19] Sholomskas DE Syracuse-Siewert G Rounsaville BJ Ball SA Nuro KF Carroll KM. We don't train in vain: a dissemination trial of three strategies of training clinicians in cognitive-behavioral therapy. J Consult Clin Psychol. (2005) 73:106–15. doi: 10.1037/0022-006X.73.1.106, 15709837 PMC2367057

[20] Girard A Doucet A Lambert M Ouadfel S Caron G Hudon C. What is known about the role of external facilitators during the implementation of complex interventions in healthcare settings? A scoping review. BMJ Open. (2024) 14:e084883. doi: 10.1136/bmjopen-2024-084883, 38951001 PMC11328637

[21] Damschroder LJ Reardon CM Opra Widerquist MA Lowery J. The updated consolidated framework for implementation research based on user feedback. Implement Sci. (2022) 17:75. doi: 10.1186/s13012-022-01245-0 PMC9617234 36309746

[22] McGinty EE Daumit GL. Integrating mental health and addiction treatment into general medical care: the role of policy. Psychiatr Serv. (2020) 71:1163–9. doi: 10.1176/appi.ps.202000183, 32487007 PMC7606646

[23] Institute of Medicine (US). Committee on Crossing the Quality Chasm: Adaptation to Mental Health and Addictive Disorders. Improving the Quality of Health Care for Mental and Substance-Use Conditions: Quality Chasm Series. Washington, DC: National Academies Press (2006). 20669433

[24] Eby LT Burk H Maher CP. How serious of a problem is staff turnover in substance abuse treatment? A longitudinal study of actual turnover. J Subst Abus Treat. (2010) 39:264–71. doi: 10.1016/j.jsat.2010.06.009, 20675097 PMC2937083

[25] Garner BR Hunter BD Modisette KC Ihnes PC Godley SH. Treatment staff turnover in organizations implementing evidence-based practices: turnover rates and their association with client outcomes. J Subst Abus Treat. (2012) 42:134–42. doi: 10.1016/j.jsat.2011.10.015, 22154040 PMC3268938

[26] Knight DK Becan JE Flynn PM. The impact of staff turnover on workplace demands and coworker relationships. Counselor. (2013) 14:20–3. PMC4986917 27540331

[27] Hath-Maillette MA Harwick R Baer JS Masters T Cloud K Peavy M . Counselor turnover in substance use disorder treatment research: observations from one multisite trial. Subst Abus. (2019) 40:214–20. doi: 10.1080/08897077.2019.157 PMC6759413 30829142

[28] Pascoe KM Petrescu-Prahova M Steinman L Bacci J Mahorter S Belza B . Exploring the impact of workforce turnover on the sustainability of evidence-based programs: a scoping review. Implement Res Pract. (2021) 2. doi: 10.1177/26334895211034581 PMC9981891 37090007

[29] Garner BR Hunter BD. Predictors of staff turnover and turnover intentions within addiction treatment settings: change over time matters. Subst Use Res Treat. (2014) 8:63–71. doi: 10.4137/SART.S17133 PMC4196888 25336960

[30] Hallett E Simeon E Amba V Howington D McConnell KJ Zhu JM. Factors influencing turnover and attrition in the public behavioral health system workforce: qualitative study. Psychiatr Serv. (2024) 75:55–63. doi: 10.1176/appi.ps.20220516, 37386878 PMC10756926

[31] Young S. Understanding substance abuse counselor turnover due to burnout: a theoretical perspective. J Hum Behav Soc Environ. (2015) 25:675–86. doi: 10.1080/10911359.2015.1013658

[32] Eby LT Laschober TC Curtis SL Sauer JB. Predictors of staff turnover and turnover intentions within addiction treatment settings: change over time matters. Substance Abuse. (2014) 8:69–79. 10.4137/SART.S17133 PMC4196888 25336960

[33] Brabson LA Harris JL Lindhiem O Herschell AD. Workforce turnover in community behavioral health agencies in the USA: a systematic review with recommendations. Clin Child Fam Psychol Rev. (2020) 23:297–315. doi: 10.1007/s10567-020-00313-5, 32103361

[34] National Association of State Alcohol and Drug Abuse Directors (NASADAD). The substance use workforce crisis: drivers, challenges, and promising strategies (2025). 1–25. Available online at: https://nasadad.org/2025/04/substance-use-workforce-crisis-drivers-challenges-and-promising-strategies/

[35] Cross-Technology Transfer Center (TTC) Workgroup on Virtual Learning. Providing behavioral workforce development technical assistance during COVID-19: adjustments and needs. Transl Behav Med. (2022) 12:180–5. doi: 10.1093/tbm/ibab097, 34409456 PMC8499729

[36] Kruger DJ Kirk HM Leonard KE Lynch JJ Nielsen N Collins RL . Assessing experts’ perspectives on challenges in substance misuse prevention, harm reduction, and treatment to shape funding priorities in New York state. Harm Reduct J. (2024) 21:Article 134. doi: 10.1186/s12954-024-01045-3, 39004729 PMC11247824

[37] Knopf A. ‘Behavioral health workforce’ will have shortage of addiction professionals: HRSA. Alcohol Drug Abuse Wkly. (2023) 35:1–2. doi: 10.1002/adaw.33764

[38] Silverman K Holtyn AF Morrison R. The therapeutic utility of employment in treating drug addiction: science to application. Transl Issues Psychol Sci. (2016) 2:203–12. doi: 10.1037/tps0000061, 27777966 PMC5074553

[39] Galfano S. Addictions treatment professional career and salary survey. Counselor. (2004) 5:24–9.

[40] Addiction Technology Transfer Center Network. Strategic response to behavioral workforce shortages (2024). 1–12. Available online at: https://attcnetwork.org/products_and_resources/clinical-supervision-foundations-course/ (Accessed May 18, 2026).

[41] Harris-Lemak C Alexander JA Campbell C. Administrative burden and its implications for outpatient substance abuse treatment organizations. Psychiatr Serv. (2003) 54:705–11. doi: 10.1176/appi.ps.54.5.705 12719502

[42] Joukes E Abu-Hanna A Cornet R de Keizer NF. Time spent on dedicated patient care and documentation tasks before and after the introduction of a structured and standardized electronic health record. Appl Clin Inform. (2018) 9:46–53. doi: 10.1055/s-0037-1615747, 29342479 PMC5801881

[43] National Council for Mental Wellbeing. New study: behavioral health workforce shortage will negatively impact society (2023). Available online at: https://www.thenationalcouncil.org/news/help-wanted/ (Accessed May 23, 2026).

[44] American Medical Association. De-implementation checklist (2021). 1–2. Available online at: https://www.ama-assn.org/system/files/de-implementation-checklist.pdf (Accessed May 23, 2026).

[45] American Medical Association. Reducing prior authorization burdens. National Advocacy Conference. (2022). 1–2. Available online at: https://www.ama-assn.org/system/files/2022-nac-action-kit-prior-auth.pdf (Accessed May 23, 2026).

[46] Melnick E Dyrbye L Sinsky C Trockel M West C Nedelec L . The association between perceived electronic health record usability and professional burnout among US physicians. Mayo Clin Proc. (2020) 95:476–87. doi: 10.1016/j.mayocp.2019.09.024 31735343

[47] Shah T Kitts AB Gold J Horvath K Ommaya A Opelka F . EHR Optimization and Clinician well-Being: A Potential Roadmap toward action. NAM Perspectives. Discussion Paper, National Academy of Medicine. Washington DC: National Academy of Medicine. (2020). 10.31478/202008a PMC8916811 35291737

[48] AAFP Innovation Labs. Using an AI assistant to reduce documentation burden in family medicine (2021). Available online at: https://www.wpchange.org/resources/using-an-ai-assistant-to-reduce-documentation-burden-in-family-medicine (Accessed May 29, 2026).

[49] American Medical Association, American Hospital Association, America's Health Insurance Plans, American Pharmacists Association, Blue Cross Blue Shield Association, Medical Group Management Association. Consensus statement on improving the prior authorization process (2018). 1–4. Available online at: https://www.ama-assn.org/sites/ama-assn.org/files/corp/media-browser/public/arc-public/prior-authorization-consensus-statement.pdf (Accessed May 29, 2026).

[50] Cook KE Ludens GM Ghosh AK Mundell WC Fleming KC Majka AJ. Improving efficiency and reducing administrative burden through electronic communication. Perm J. (2013) 17:26–30. doi: 10.7812/TPP/12-010, 23596365 PMC3627784

[51] Ashton M. Getting rid of stupid stuff. N Engl J Med. (2018) 379:1789–91. doi: 10.1056/NEJMp1809698 30403948

[52] Kelly L. Burnout, compassion fatigue, and secondary trauma in nurses: recognizing the occupational phenomenon and personal consequences of caregiving. Crit Care Nurs Q. (2020) 43:73–80. doi: 10.1097/CNQ.0000000000000293 31789880

[53] Slatten LA Carson KD Carson PP. Compassion fatigue and burnout: what managers should know. Health Care Manag. (2020) 39:181–9. doi: 10.1097/HCM.0b013e31823511f7 33079770

[54] Grebski M Mazur M. Management strategies to avoid professional burnout. Pol J Manag Stud. (2022) 26:61–75. doi: 10.17512/pjms.2022.26.1.04

[55] Nashwan AJ. The vital role of career pathways in nursing: a key to growth and retention. Cureus. (2023) 15:1–2. doi: 10.7759/cureus.38834, 37303402 PMC10254089

[56] Ahmed A. Rethinking employee retention: the role of career path transparency in reducing turnover. International Journal of Research in Human Resource Management. (2024) 6:450–6. doi: 10.33545/26633213.2024.v6.i2e.337

[57] Becker SJ Kemo M Bailey A Scott K Zelaya DG Kearns MDH . Cultivating leaders in the addiction behavioral health workforce: New England addiction technology transfer center's leadership development program. Subst Use Addctn J. (2026) 47:645–55. doi: 10.1177/29767342251392341, 41784141

[58] Rothwell C Kehoe A Farook SF Illing J. Enablers and barriers to effective clinical supervision in the workplace: a rapid evidence review. BMJ Open. (2021) 11:e052929. doi: 10.1136/bmjopen-2021-052929, 34588261 PMC8479981

[59] Martin P Lizarondo L Kumar S Snowdon D. Impact of clinical supervision on healthcare organisational outcomes: a mixed methods systematic review. PLoS One. (2021) 16:e0260156. doi: 10.1371/journal.pone.0260156, 34797897 PMC8604366

[60] Addiction Technology Transfer Center Network. Clinical supervision foundations course (2024). Available online at: https://attcnetwork.org/products_and_resources/clinical-supervision-foundations-course/ (Accessed May 29, 2026).

